# Molecular and Physiological Responses of Plants that Enhance Cold Tolerance

**DOI:** 10.3390/ijms26031157

**Published:** 2025-01-29

**Authors:** Lixia Zhou, Fazal Ullah, Jixin Zou, Xianhai Zeng

**Affiliations:** 1 National Key Laboratory for Tropical Crop Breeding, Chinese Academy of Tropical Agricultural Sciences, Haikou 571101, China; lxzhou@catas.cn; 2 Hainan Key Laboratory of Tropical Oil Crops Biology, Coconut Research Institute, Chinese Academy of Tropical Agricultural Sciences, Wenchang 571339, China; 3 College of Life Sciences, Northwest Normal University, Lanzhou 730070, China; fazalbiologist@gmail.com

**Keywords:** cold acclimation, CBF, ICE1, molecular mechanism, plant growth and development

## Abstract

Low-temperature stress, including chilling and freezing injuries, significantly impacts plant growth in tropical and temperate regions. Plants respond to cold stress by activating mechanisms that enhance freezing tolerance, such as regulating photosynthesis, metabolism, and protein pathways and producing osmotic regulators and antioxidants. Membrane stability is crucial, with cold-resistant plants exhibiting higher lipid unsaturation to maintain fluidity and normal metabolism. Low temperatures disrupt reactive oxygen species (ROS) metabolism, leading to oxidative damage, which is mitigated by antioxidant defenses. Hormonal regulation, involving ABA, auxin, gibberellins, and others, further supports cold adaptation. Plants also manage osmotic balance by accumulating osmotic regulators like proline and sugars. Through complex regulatory pathways, including the ICE1-CBF-COR cascade, plants optimize gene expression to survive cold stress, ensuring adaptability to freezing conditions. This study reviews the recent advancements in genetic engineering technologies aimed at enhancing the cold resistance of agricultural crops. The goal is to provide insights for further improving plant cold tolerance and developing new cold-tolerant varieties.

## 1. Introduction

During the growth process, plants are often affected by various abiotic stresses, such as cold, heat, drought, heavy metal, and salt stresses [1,2,3]. These abiotic stresses limit the geographical distribution range of plants and reduce their productivity [4]. Low-temperature stress has a great impact on plants. It will damage plants’ plasma membrane structure, reduce plants’ photosynthetic capacity, cause an excessive accumulation of reactive oxygen species (ROS), and lead to phenomena such as a slowdown and even stagnation of plant growth and development [5,6].

Over the long course of evolution, plants have developed a series of complex and efficient network regulation mechanisms to cope with the threats posed by low temperatures [7]. With the continuous development of molecular biology technology, the mechanisms by which plants respond to cold stress have gradually become clear. At the physiological and biochemical levels, plants produce a series of osmoregulatory substances such as soluble sugars, proline, and polyamine compounds to stabilize the cell membrane structure and eliminate ROS [8]. In addition, many protein kinases and transcription factors play a role in the cold stress signal pathway. CBF/DREB1 (C-Repeat Binding Factor/Dehydration-Responsive Element-Binding Protein 1) is the most critical regulator [9]. Under cold stress, CBF genes are induced by upstream ICE1 (Inducer of CBF Expression 1) to be highly expressed, and they then catalyze the expression of cold-regulated (COR) genes to regulate cold stress [10]. Compared with the CBF-dependent signaling pathway, the non-CBF-dependent signaling pathway also plays an important role [11,12]. For example, the Sfr6 (Sensitive to freezing 6) gene directly regulates the expression of downstream COR genes without inducing CBF genes. At the same time, cold stress-related genes also play a key role in transcription, translation, and post-translational modifications [13]. This review synthesizes recent research on the regulatory mechanisms underlying plant responses to low-temperature stress. It outlines current advancements in genetic engineering techniques to enhance the cold resistance of economically important crops. The objective is to provide a theoretical foundation for further investigations into plant responses to low-temperature stress and to support the development of new varieties with improved cold tolerance.

## 2. Plant Physiological Responses to Low-Temperature Stress

After being subjected to low-temperature stress, various physiological and biochemical indicators and plant organelle morphology will undergo significant changes [14]. Under low-temperature stress, many physiological and biochemical functions of plant cells will change. This includes changes in cell membranes and lipids, such as the leakage of ions and amino acids within cells and structural changes in various cell components. Ultimately, these lead to plant necrosis or death [15,16].

### 2.1. Impact of Low Temperature on Plant Cell Membrane Stability and Metabolism

The cell membrane, also known as the plasma membrane, is composed of a phospholipid bilayer, proteins, and a small amount of carbohydrates. The proteins are embedded in the phospholipid bilayer. Lipids combine with carbohydrates to form glycolipids, and proteins combine with carbohydrates to form glycoproteins [17]. The cell membrane has important physiological functions. It can maintain the intracellular environment, isolating plant cells from the external environment. Meanwhile, it enables the cells to exchange substances and transmit information with the external environment. Studies have shown that the cell membrane is the first to sense low-temperature stress and respond accordingly [18,19]. Low-temperature stress can cause a phase transition in the cell membrane, i.e., from a liquid crystalline state to a gel state. At the same time, proteins are denatured, and the disordered fatty acid chains become ordered, while the membrane permeability increases and the electrolyte balance is disrupted [20]. Under normal circumstances, there are some enzymes attached to the cell membrane. Thus, the phase transition of the plasma membrane under low temperature causes these enzymes to be released and lose their activity. As a result, ATP synthesis is inhibited, and the coupling between photophosphorylation and oxidative phosphorylation uncouples, ultimately leading to metabolic disorders. Consequently, in severe cases, the plants may die [19]. The degree of membrane lipid unsaturation determines the stability of the cell membrane. The higher the degree of unsaturation, the weaker the membrane stability. Plants with stronger cold resistance have a higher degree of membrane lipid unsaturation, which enables them to maintain the fluidity of the cell membrane and normal physiological metabolism even at lower temperatures. When subjected to low-temperature stress, many plants will produce more fatty acids with a higher degree of unsaturation (such as linolenic acid, oleic acid, and linoleic acid) to resist low temperatures. The degree of membrane lipid unsaturation in plants is determined by genetic stability on the one hand and can be enhanced by low-temperature hardening and induction on the other hand [21]. Zhang et al. found that *OsKASI-2* was a critical gene for regulating the degree of unsaturation in membrane lipids for the maintenance of the membrane structural homeostasis of rice under cold stress [22].

### 2.2. Impact of Low Temperature on ROS Metabolism and Antioxidant Defense in Plants

Low temperature inhibits the normal reactive oxygen species (ROS) metabolism in plants. The ROS produced by plant metabolism mainly include hydrogen peroxide (H_2_O_2_), hydroxide ion (OH^−^), hydroxyl radical (OH), superoxide anion (O_2_^−^), etc. [23]. ROS are mainly produced by cell membranes, chloroplasts, mitochondria, and peroxisomes, among which chloroplasts are the main production sites. During the normal growth of plants, ROS are very important signaling molecules that participate in the molecular, physiological, and biochemical reactions of plant cells. For example, they can take part in the defense responses and programmed cell death processes of plants, playing crucial roles in maintaining plant homeostasis [24]. However, ROS are highly toxic, and the excessive accumulation of ROS can be toxic to intracellular proteins, carbohydrates, and genetic materials, thereby inhibiting the normal growth of plants. Therefore, normal plants possess a complete set of antioxidant defense mechanisms that can remove excess ROS molecules in cells at any time [25]. The antioxidant defense mechanism in higher plants is composed of many antioxidant enzymes and non-enzymatic antioxidants. The antioxidant enzymes include superoxide dismutase (SOD), peroxidase (POD), catalase (CAT), ascorbate reductase (APX), glutathione reductase (GR), etc.; the non-enzymatic antioxidants mainly include ascorbic acid (ASA) and glutathione (GSH), etc. The ability of plants to resist external stress is correlated with the level of the antioxidant system [26].

SOD, POD, and CAT are all very important antioxidant enzymes. Among them, SOD is the first enzyme involved in antioxidant action. Its main function is to scavenge superoxide anion (O_2_^−^) while producing H_2_O_2_ at the same time. POD and CAT degrade excess ROS such as H_2_O_2_ through enzymatic action, preventing plants from peroxidative damage. POD not only has the function of scavenging ROS, but its main functions are also related to lignin synthesis and cell disease resistance [27]. Many studies have shown that under low-temperature stress, the activities of SOD, POD, and CAT in cucumber [28], maize [29], rice [30], etc., show a trend of first increasing and then decreasing with the decrease in stress temperature. This indicates that low temperature can induce the increase in antioxidant enzyme activities, thereby alleviating the damage caused by low temperature. However, as the stress effect continues to intensify, the antioxidant defense system is damaged, the enzyme activity decreases, and the membrane lipid peroxidation is enhanced. Some studies have shown that among the three antioxidant enzymes, SOD and CAT are more sensitive to low temperature, and their activities increase rapidly, while the activity of POD changes little or even decreases slightly. This may be an active coping strategy exhibited by plants to adapt to adverse environments. When a certain or some metabolic processes are enhanced, other metabolic pathways may be inhibited. Research by Zeng et al., 2023, found that the rates of increase and decrease in the enzyme activities of SOD, POD, and CAT are very closely related to the cold tolerance of plants [31].

Another enzymatic catalytic system for plants to scavenge ROS is the ascorbic acid (ASA)–glutathione (GSH) cycle, which mainly exists in chloroplasts, mitochondria, and cytoplasm. Ascorbate peroxidase (APX), ascorbic acid (ASA), glutathione reductase (GR), and glutathione (GSH) are important components of this cycle [32]. APX is one of the key enzymes for scavenging hydrogen peroxide. It mainly exists in chloroplasts, where POD and CAT enzymes are absent. Therefore, APX is the key enzyme for scavenging H_2_O_2_ in chloroplasts. According to the location of APX in chloroplasts, APX can be divided into four types: thylakoid membrane APX (tAPX), chloroplast stroma APX (sAPX), cytoplasmic APX (cAPX), and microbody APX (mbAPX) [33]. Xu et al., 2022, treated two rice cultivars with different cold resistance using eight different low-temperature treatments, with 18 °C serving as the control. The results showed that the activity of APX in the apricot cultivar with strong resistance to late frost was the highest compared with the control under low-temperature stress [34]. Li et al., 2019, studied the low-temperature stress of tea and found that the activity of APX was positively correlated with the content of H_2_O_2_ and played a very important role in the process of scavenging H_2_O_2_ [35].

### 2.3. Role of Osmotic Regulators in Enhancing Cold Tolerance in Plants

When plants are exposed to low temperatures, water loss occurs in cells. At this time, some plants will actively accumulate various organic or inorganic substances in their bodies to lower the osmotic potential of cells, prevent excessive water loss from plant cells, and maintain a certain osmotic concentration in cells. The main osmotic adjustment substances in plants are divided into two categories: inorganic ions and organic solutes. Specifically, they include proline, soluble sugars, soluble proteins, and polyamine [36].

During the period affected by low-temperature stress, the level of soluble sugars in plant cells will increase to some extent. Soluble sugars act as osmoprotectants in plants. When they accumulate in cells, the osmotic concentration increases, alleviating the cell shrinking effect caused by low temperature, thereby protecting cell membranes from dehydration and freezing damage [37,38]. Li et al., 2024, found that glucose irrigation mainly promoted soluble sugar accumulation to reduce cold damage in melon seedlings [39]. Yang et al., 2020, identified hypoploid *Saccharum spontaneum* enhanced its osmotic adjustment system through sugar accumulation, thereby increasing its cold tolerance [38]. In plants, proline also plays an important role in osmotic regulation. It helps maintain hydrophobic interactions critical for protein function, supports proper protein folding, and stabilizes protein structures [39]. Proline’s unique cyclic structure enables it to act as a molecular chaperone, preventing protein denaturation and aggregation during osmotic stress [40]. Additionally, proline stabilizes polyribosomes, which are essential for efficient protein synthesis under stress [41]. Research indicates that proline accumulation enhances ribosome activity, promoting the translation of stress-responsive proteins [42]. This dual role in protein production and stabilization underscores proline’s significance in plant stress tolerance mechanisms. Peppino Margutti et al., 2024, found that the accumulation of proline enhanced the cold stress tolerance of barley [43]. In short, these osmotic regulators resist the adverse effects of low-temperature stress by maintaining the balance of intracellular osmotic levels. Meanwhile, when suffering from low-temperature stress, a large amount of reactive oxygen species (ROS) will be generated in plants [24]. ROS are mainly produced in plant cells through electron transfer in chloroplasts and mitochondria and enzymatic reactions in peroxisomes and apoplasts. Superoxide anion radical (O_2_^−^), hydroxyl radical (OH), and hydrogen peroxide (H_2_O_2_) all belong to the category of ROS [44]. Under low-temperature stress conditions, excessive ROS are produced in plants, leading to protein denaturation, lipid peroxidation, and nucleotide degradation, causing cell damage and, ultimately, cell death [45]. These results indicate that ROS are one of the important reasons for plant damage caused by low-temperature stress. Polyamines are aliphatic nitrogenous bases exhibiting biological activity. They play diverse and crucial physiological roles in plants’ responses to low-temperature stress. Polyamines function as osmotic regulators. They increase the solute concentration within the cell, thereby lowering the cell water potential and enhancing the water holding capacity of plant cells. In a low-temperature environment, plant cells are prone to water loss. The accumulation of polyamines aids in maintaining cell turgor pressure and normal physiological functions. This enables cells to maintain a certain water balance under low-temperature stress and reduces damage caused by dehydration. For instance, in rice seedlings subjected to low-temperature treatment, the polyamine content in the seedlings increased, which helped the seedlings retain intracellular water and enhanced their low-temperature tolerance [46]. Polyamines carry a positive charge and can interact with negatively charged biomacromolecules such as proteins and nucleic acids. This charge-based interaction is particularly crucial under low-temperature stress. During low-temperature stress, polyamines can stabilize the structures of proteins and nucleic acids through these interactions, preventing them from denaturing or degrading due to low temperature and ensuring the normal physiological functions of biomacromolecules. For example, polyamines can bind to DNA, safeguarding its double-helix structure and maintaining the normal progress of gene expression and replication. Biomembranes are vulnerable to damage at low temperatures, which can lead to a decrease in membrane fluidity and changes in permeability [47]. Polyamines can be inserted into the phospholipid bilayer of biomembranes, regulate the fluidity of membrane lipids and the activity of membrane proteins, enhance the stability of biomembranes, reduce membrane damage caused by low temperature, maintain the normal functions of membranes, and ensure the smooth progress of physiological processes such as intracellular substance transport and signal transduction. Polyamines can increase the activity of antioxidant enzymes in plants, such as superoxide dismutase (SOD), peroxidase (POD), and catalase (CAT), which can scavenge excessive reactive oxygen species (ROS) generated in cells under low-temperature stress, such as superoxide anions and hydrogen peroxide, and reduce the damage caused by oxidative stress to cells. For example, in tomato plants under low-temperature stress, treatment with polyamines can significantly increase the activity of antioxidant enzymes, reduce ROS accumulation, and alleviate the damage of low temperature to the plants [48]. Polyamines themselves also possess a certain antioxidant capacity and can directly react with reactive oxygen species, converting them into harmless or less toxic substances. This reduces the oxidative damage of reactive oxygen species to biomacromolecules and membrane structures inside the cell and protects cells from oxidative stress induced by low temperature. There is a close interaction between polyamines and plant hormones, which jointly regulate the plant’s response to low-temperature stress. For example, polyamines can influence the synthesis and signal transduction of abscisic acid (ABA). Under low-temperature stress, changes in polyamine levels may promote the synthesis of ABA or enhance the plant’s sensitivity to ABA. Subsequently, through the ABA signaling pathway, the plant’s physiological responses, such as promoting stomatal closure and inducing the expression of low temperature response genes, are regulated, thereby improving the plant’s tolerance to low temperature. In addition, polyamines also interact with hormones such as auxin and cytokinin, coordinately regulating the growth and development of plants in a low-temperature environment [49]. Polyamines can regulate gene expression by binding to chromatin. Under low-temperature stress, polyamines can induce or inhibit the expression of certain genes related to the low temperature response, thereby regulating a series of physiological and biochemical processes in plants to adapt to the low temperature environment. For example, polyamines may promote the expression of some genes encoding low-temperature-induced proteins, and these proteins help plants enhance their resistance to low temperature [48].

### 2.4. Regulatory Roles of Plant Hormones During Low-Temperature Stress

Plant hormones play important regulatory roles during low-temperature stress. Different types of plant hormones help plants cope with low-temperature environments through their unique pathways and mechanisms. The regulatory roles of several common plant hormones under low-temperature stress are described below.

#### 2.4.1. Abscisic Acid (ABA)

ABA is one of the key hormones through which plants cope with low-temperature stress. In a low-temperature environment, the content of ABA in plants increases rapidly. ABA regulates the ion channels in guard cells, causing guard cells to lose water and thus inducing stomatal closure [39]. For example, under stress conditions such as drought and low temperature, ABA promotes the efflux of ions such as K^+^, resulting in a decrease in the turgor pressure of guard cells, stomatal closure, reduced water loss, and enhanced water-holding capacity of plants, which helps maintain the water balance of cells and prevents cells from being damaged due to water loss [50]. ABA can also induce the expression of a series of genes related to cold resistance. The products of these genes include some protective proteins and enzymes related to the synthesis of osmoregulatory substances. For example, ABA can induce the expression of some genes encoding dehydrins. Dehydrins can stabilize the cell membrane and protein structure, enhancing the cold resistance of cells [51]. In addition, ABA can promote the synthesis of osmoregulatory substances such as proline, reducing the osmotic potential of cells and preventing excessive water loss of cells at low temperatures [52].

#### 2.4.2. Auxin (IAA)

Under low-temperature stress, the distribution and transport of auxin change. Auxin can maintain the growth and differentiation of plant cells. Under low-temperature conditions, it maintains the normal growth of cells by regulating the extensibility of the cell wall and the turgor pressure inside the cell [53]. For example, auxin can promote the transport of protons to the cell wall, acidifying the cell wall and increasing its extensibility, so that cells can continue to grow at low temperatures. Auxin has an important impact on root development. Under low-temperature stress, auxin promotes the growth and differentiation of roots, increasing the absorption area and efficiency of roots. For example, auxin can induce the formation of lateral roots, making the root system more developed, thereby enhancing the plant’s ability to absorb water and nutrients and providing guarantee for the growth of plants in a low-temperature environment [54].

#### 2.4.3. Gibberellin (GA)

Gibberellin plays an important role in the growth and development of plants. Under low-temperature stress, the synthesis and signal transduction pathways of GA are affected. On the one hand, GA can promote the elongation of plant stems and the expansion of leaves, enabling plants to maintain a certain growth trend at low temperatures. On the other hand, GA can also regulate the development process of plants, such as promoting seed germination and flower bud differentiation. For example, in some plants, an appropriate concentration of GA can break seed dormancy and promote seed germination in a low-temperature environment. There are complex interactions between GA and other plant hormones. Under low-temperature stress, the balance between GA and ABA has an important impact on the cold resistance of plants. For example, when the content of ABA increases, the synthesis and activity of GA may be inhibited, thus regulating the growth and development process of plants to a certain extent to adapt to the low-temperature environment [55].

#### 2.4.4. Cytokinin (CTK)

Cytokinin is mainly synthesized in actively growing parts of plants such as the root tip and shoot tip. Under low-temperature stress, CTK can promote cell division and differentiation, maintaining the growth and development of plants. For example, CTK can induce the expression of genes related to the cell cycle, promoting cells to enter the S phase from the G_1_ phase, thereby increasing the cell division frequency. In addition, CTK can also promote bud differentiation and lateral branch growth, enabling plants to maintain a certain growth vitality at low temperatures. CTK has the effect of delaying leaf senescence. Under low-temperature stress, leaves are vulnerable to damage and senescence. CTK can delay leaf senescence by regulating physiological processes in leaves, such as photosynthesis and protein synthesis. For example, CTK can increase the chlorophyll content in leaves, maintaining the normal progress of photosynthesis and providing more energy and material basis for plants [56].

#### 2.4.5. Ethylene (ETH)

Ethylene is a gaseous plant hormone with various physiological functions in the growth and development of plants. Under low-temperature stress, the synthesis and signal transduction of ethylene change. Ethylene can regulate plant physiological processes, such as promoting fruit ripening and leaf abscission. In a low-temperature environment, ethylene may regulate the growth and development process of plants to adapt to low-temperature stress [57]. For example, ethylene can induce the expression of some genes related to cold resistance, enhancing the cold resistance of plants. Ethylene also participates in plant defense responses. Under low-temperature stress, ethylene can induce plants to produce some defense substances, such as phytoalexins and pathogenesis-related proteins, enhancing the plant’s resistance to pathogens. For example, ethylene can induce plants to synthesize some secondary metabolites with antibacterial activity, thereby reducing pathogen infection and protecting plants from the dual damage of low temperature and pathogens [58].

#### 2.4.6. Jasmonic Acid (JA)

Jasmonic acid plays an important role in plant defense responses. Under low-temperature stress, JA can induce the expression of a series of defense genes. The products of these genes include some antioxidant enzymes and heat shock proteins. For example, JA can induce the synthesis of antioxidant enzymes such as superoxide dismutase (SOD) and peroxidase (POD), scavenging excessive reactive oxygen species (ROS) in plants and reducing the oxidative damage of cells caused by low-temperature stress. JA can also regulate plant growth and development. Under low-temperature stress, JA can inhibit plant growth, enabling plants to allocate more energy to defense responses. For example, JA can inhibit the elongation of plant stems and the expansion of leaves, reducing plant growth consumption and thus improving the cold resistance of plants [59].

## 3. Molecular Mechanisms of Cold Acclimation

During the long-term growth and development process, plants have evolved a series of complex network regulatory mechanisms to cope with low-temperature stress, mainly including the CBF-dependent signaling pathway and the non-CBF-dependent signaling pathway [60].

### 3.1. CBF-Dependent Mechanism

At present, many studies have explored the regulatory mechanisms of plants in response to cold. The CBF pathway is the most crucial regulatory pathway for plants to cope with low-temperature stress. The ICE1 (Inducer of CBF Expression 1)-CBF (C-Repeat Binding Factor)-COR (Cold Regulated) signal transduction cascade is the most important low-temperature signal pathway among them [61]. Under low-temperature stress conditions, the transcription factor ICE1 binds to the promoter regions of CBF genes and activates the expression of CBFs, and the activated CBFs further activate the expression of downstream COR genes, thereby enhancing the cold resistance of plants [62].

#### 3.1.1. ICE1-CBF-COR Regulatory Pathway

C-Repeat Binding Factors/Dehydration Responsive Element Binding Protein (CBF/DREB) is the most typical transcription factor for regulating low-temperature stress signals. It belongs to a subfamily in the ERF family of the AP2/ERF super protein family, which plays a role in plant growth, development, and abiotic stress responses and contains only one AP2 domain [63]. Its members—*CBF1*, *CBF2*, and *CBF3* (*DREB1B*, *DREB1C*, *DREB1A*)—have been proven to be involved in the low-temperature stress response process and can be induced within a short time when plants are under low-temperature conditions [64]. Studies have shown that the three CBF proteins have a very high (86%) sequence similarity. Although cold responses induce all of them, there is still functional redundancy among these three CBF proteins [65]. The CBF1 and CBF3 proteins are more similar in function, while the CBF2 protein is different in its expression pattern than the other two CBFs [66]. In the early development stage, CBF1 and CBF3 are specifically expressed in the roots, hypocotyls, and cotyledons of plants. In contrast, CBF2 is expressed in the hypocotyls and cotyledons and cannot be expressed in the roots. Some studies have proven that under *CBF2* mutation, the expression levels of the *CBF1* and *CBF3* genes increase, showing an increased tolerance to freezing, indicating that *CBF2* is a negative regulator of CBF1 and CBF3 expression [67].

The CBF gene binds to the CRT/DRE cis-element (A/GCCGAC) existing in the promoters of downstream target genes through its AP2/ERF DNA binding domain and activates the transcription of downstream COR genes to increase cold tolerance [68]. After being regulated, cold-regulated (COR) genes will generate some antifreeze polypeptides, transcription factors, protein kinases, proteins related to embryogenesis and lipid metabolism, proteins related to hormone responses and cell wall modification, etc. These products can help plants stabilize the structure of cell membranes under low-temperature stress, thereby enhancing their cold tolerance [69].

Upstream of the cold response cascade pathway is a member of the *bHLH* transcription factor family, *ICE1* (Inducer of CBF Expression 1). It is a MYC-type basic helix–loop–helix (bHLH) transcription factor. It contains a conserved Bhlh binding domain at the C terminus, which is used to bind to the typical MYC *cis*-*element* (CANNTG) in the promoter of the downstream *CBF3*/*DREB1A*, positively inducing the expression of the *CBF3*/*DREB1A* regulator. At the same time, *CBF1* and *CBF2* are not affected [70]. The homolog of *ICE1*, *ICE2*, has a high degree of sequence identity with *ICE1* and encodes the same bHLH domain sequence. ICE1 and ICE2 are major regulators of stomatal formation and play important roles in regulating cold responses [71]. Current research shows that *ICE1* and *ICE2* have functional redundancy and different ways of inducing *CBF* genes. *ICE1* and *ICE2* regulate the expressions of *CBF3* and *CBF1*, respectively, by inducing *CBF2* and reducing the freezing tolerance of plants [72].

#### 3.1.2. Positive Regulation in CBF Transcriptional Regulation

The process of the low-temperature stress response involves a series of transcriptional pathways, and some constitutively expressed transcription factors can be activated in response to cold. Experiments have proven that these transcription factors can positively induce the transcription of downstream *CBF* genes. For example, *ICE1* is a positive regulator of *CBFs*. It can bind to the MYC recognition site in the promoter of the downstream gene CBF3 to regulate *CBF3* expression. In the ice1 mutant, the expression ability of *CBF3* is reduced. Compared with the wild type, the mutant has greatly reduced cold tolerance and cannot be cold acclimated [50]. The overexpression of *ICE1* greatly increases the expressions of the *CBF1*, *CBF2*, *CBF3*, and *COR* genes, enhancing the plant’s freezing resistance [73].

The calmodulin-binding transcriptional activator (CAMTA) involved in signal transduction is also a positive regulator of CBF [74]. It can respond to a rapid drop in temperature. It has a CG-1 domain with specific DNA-binding activity, which can positively regulate the CM2 element in the CBF2 promoter and strongly induce the expressions of CBF1 and CBF2 [74]. *Brassina Zole Resistant 1* (*BZR1*), a key transcription factor in the brassinosteroid signaling pathway, binds to the BRRE (CGTGT/CG) and E-box (CACGTG and CACTTG) motifs in the promoters of target genes, promotes the expressions of genes encoding *CBFs*, *WRKY6*, *WRKY54*, and the abscisic acid (ABA) receptor *PYL6*, and positively regulates the cold tolerance of plants [75]. Meanwhile, the protein level and phosphorylation state of BZR1 depend on the influence of upstream GSK3 like kinase BIN2 (BR-Insensitive 2) in brassinosteroid (BR) signal transduction. Without BRs, the active BIN2 phosphorylates BZR1 and promotes its degradation, increasing the plant’s freezing sensitivity [76].

#### 3.1.3. Negative Regulatory Roles in CBF Transcriptional Regulation

There are multiple negative regulators in the signaling pathway that reduce the freezing tolerance of plants, alongside the positive regulatory mechanisms. The MYB transcription factor family member *MYB15* has been proven to interact with *ICE1* and bind to *CBF* promoter elements. Under cold stress, *MYB15* expression is upregulated. Overexpression of *MYB15* shows a decrease in *CBF* gene transcription levels, while in the MYB15 mutant, the levels of *CBF3*, *CBF1*, and *CBF2* increase, indicating that *MYB15* is involved in the cold regulation of *CBF* genes and reduces the plant’s cold stress tolerance [77]. The C2H2 zinc finger protein gene *ZAT12* (*Zinc Finger Transcription Factor 12*) regulates cold acclimation by suppressing the expression of 15 cold-responsive genes and activating the expression of 9 cold-responsive genes. *ZAT12* also downregulates the expression of *CBFs* genes, indicating that it plays a negative regulatory role in plant adaptation to cold stress [78]. The ethylene signaling pathway’s transcription factor *EIN3* (*Ethylene-Insensitive 3*) negatively affects cold tolerance. The EIN3 protein binds to specific binding motifs in the CBF3 promoter, preventing transcription. Meanwhile, the type A regulators *ARR5*, *ARR7*, and *ARR15* in the cytokinin signaling pathway have been proven to be downstream target genes of EIN3. In transgenic plants overexpressing *EIN3*, the transcription and protein stability of *ARR5*, *ARR7*, and *ARR15* are significantly inhibited, indicating that *EIN3* exerts an antagonistic effect in cold stress responses by combining ethylene and cytokinin signaling pathways [79]. Investigating the roles of negative regulators within the CBF signaling pathway is crucial for identifying new targets to improve plant freezing tolerance. Future studies should aim to characterize the interplay between these negative regulators and other signaling pathways, as this could reveal novel approaches for enhancing cold resilience in crops under changing climatic conditions.

### 3.2. CBF Independent Mechanisms

Although *CBF* and its target genes are crucial for cold stress signaling, some genes are still independent of the CBF pathway. The increase or decrease in their functions does not affect the expression of CBF gene functions but can increase or decrease the freezing tolerance of plants. For example, *HY5* (*Elongated Hypocotyl 5*) is a *bZIP* transcription factor in light signal transduction. When affected by low-temperature stress, the E3 ubiquitin ligase COP1 (Constitutively Photomorphogenic 1) promotes the stable expression of HY5, positively regulating the expression of downstream COR genes through CBF-independent pathways [80]. The SFR6 (Sensitive to Freezing 6) gene has been proven to regulate downstream COR genes through the CRT/DRE promoter sequence motif. In the freezing-sensitive mutant sfr6, the expression levels of COR genes, including *KIN1*/2, *LTI78*, *COR15A*, etc., are significantly reduced [81]. However, in the sfr6 mutant, the cold-induced expression of *CBF1*, *CBF2*, and *CBF3* is unaffected. As a constitutively expressed gene, the expression of *HOS9* (*High Expression of Osmotically Responsive Gene 9*) is induced under cold conditions, significantly increasing the transcription levels of *RD29A* and some other *COR* genes compared to the wild type. In the mutant *hos9-1*, the expression of CBF transcription factor genes is not affected, yet it still exhibits sensitivity to freezing stress [82].

### 3.3. Post-Transcriptional Regulation

Low-temperature stress induces extensive post-transcriptional and post-translational modifications (PTM) in plants, which affect the quality and quantity of generated mRNA, change gene functions, and ultimately influence plants’ low-temperature stress tolerance. Post-transcriptional regulation plays a crucial role in the cold acclimation process. For example, *STA1* (*Stabilized 1*), a splicing factor for mRNA precursors, is involved in mRNA splicing to remove introns, enabling normal gene expression. However, the sta1-1 mutant has defects in splicing the cold induced *COR15A* gene, which prevents the *COR15A* gene from being expressed normally and reduces the plant’s cold tolerance. This indicates that the expression of *STA1* plays a key role in the plant’s cold tolerance [83]. The nucleoporin NUP160 (Nucleoporin 160) is important in controlling RNA nucleocytoplasmic transport. In the *Arabidopsis nup160* mutant, the expression of *CBF3* is inhibited, and the mRNA transport process in the nucleus is disrupted, indicating that NUPl60 plays an important role in plant growth, flowering time regulation, and low-temperature stress tolerance [84]. The osmotic response factor *LOS4* (Low expression of osmotically responsive gene 4) encodes a DEAD-box RNA helicase which is involved in the RNA metabolic process. The *LOS4* mutant negatively regulates the expression of *CBFs*, reduces the expression of *RD29A* and other *COR* genes, and thus negatively regulates the plant’s cold resistance. This shows that *LOS4* is a positive regulator of the *CBF* gene and plays a key role in gene regulation and plant cold tolerance [85]. Post-transcriptional regulation is vital for plant responses to low-temperature stress, and further research should investigate the specific mechanisms and interactions of splicing factors, nucleoporins, and RNA helicases to enhance our understanding of how these processes contribute to cold tolerance in plants.

### 3.4. Post-Translational Regulation

In post-translational modification (PTM), several genes interact with *ICE*, *CBF*, and *COR* genes to alter their activity, conformation, localization, and stability. Phosphorylation, ubiquitination, and SUMO conjugation are the main PTM processes in plants that regulate the low-temperature stress response pathway [86]. When plants are subjected to cold stress, *ICE1* undergoes post-translational modifications, affecting the expression of CBF or other downstream genes.

#### 3.4.1. Phosphorylation Modification

Phosphorylation plays a significant role in plant cold adaptation and is a reversible protein modification highly dependent on kinases and phosphatases [87]. OST1 (Open Stomata 1)/SnRK2.6/SRK2E, as the most common phosphatase, is a serine/threonine (Ser/Thr) protein kinase in the ABA signaling pathway that can phosphorylate the ICE1 gene, helping to increase its transcriptional stability [88]. Additionally, the OST1 protein inhibits the degradation of ICE1 mediated by HOS1 by interfering with the binding of HOS1 to ICE1 protein, thereby enhancing the activity of CBF genes under dual actions to alleviate cold stress effects [84].

The Mitogen-Activated Protein Kinase (MAPK) cascade also participates in plant low-temperature stress responses through phosphorylation. MAPK is a group of serine-threonine protein kinases that can be activated by extracellular stimuli such as cytokines, neurotransmitters, hormones, cellular stress, and cell adhesion [89]. The cascade process consists of MAPK kinase kinase (MAPKKK), MAPK kinase (MAPKK), and MAPK. After plants perceive external signal stimuli, MAPKKKs are phosphorylated and activated, which subsequently phosphorylate MAPKKs. Activated MAPKKs then phosphorylate MAPKs, and finally, activated MAPKs phosphorylate specific downstream substrates such as transcription factors, kinases, or other enzymes, collectively regulating plant growth and development, biotic and abiotic stress responses, and plant hormone signal transduction [90]. The cold-responsive cascade includes MEKK1, MKK2, MPK4, and MPK6 kinases. Under cold stress, MEKK1 is upregulated, which activates downstream *MKK2*. *MKK2* specifically phosphorylates and activates its downstream target *MPK4*. Meanwhile, *MPK3* and *MPK6* are also activated by cold treatment. The activated *MPK3*/*MPK6* pathway promotes the degradation of ICE1 by phosphorylating at Ser94, Thr366, and Ser403 sites, thereby reducing the transcription of CBF genes to achieve negative regulation [91]. In contrast, the MEKK1-MKK2-MPK4 cascade positively regulates cold responses and inhibits the kinase activity of MPK3 and MPK6.

#### 3.4.2. Ubiquitination and SUMOylation Modifications

Ubiquitination refers to the process in which ubiquitin molecules, under the action of a series of specific enzymes, classify intracellular proteins, select specific target proteins, and carry out specific modifications on them [92]. Ubiquitination modification involves a series of enzymatic reactions linked by ubiquitin activating enzyme E1, ubiquitin conjugating enzyme E2, and ubiquitin ligase E3. E3 ubiquitin ligase plays the most important role in interacting with target molecules and providing a scaffold for the ubiquitination reaction [93]. HOS1 (High Expression of Osmotically Responsive Gene 1) is a functional ring finger protein with ubiquitin E3 ligase activity. It contains a variant RING finger domain and targets ICE1 for ubiquitin-mediated protein degradation, negatively regulates the expression of CBF, and ultimately reduces the cold tolerance of plants [94].

Another post-translational modification of proteins is SUMOylation modification. SUMO molecules are coupled with target proteins through the cascade of E1 activating enzyme, E2 conjugating enzyme, and E3 ligase, regulating the structure and function of target proteins [95]. SUMO-modifying proteins (SENPs), together with SUMO molecules, regulate the SUMOylation state of receptor proteins, specifically performing de-SUMOylation modification on substrate proteins, and then changing cell functions [96].

**Figure 1:**
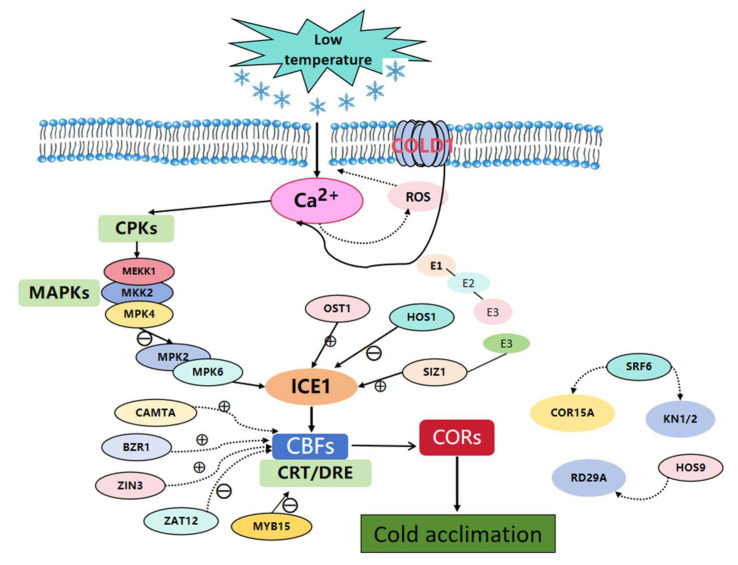
Low temperature response signal transduction pathway of plants. ⊕ means promoting effect and Θ means inhibitory effect.

SUMOylation modification plays an important role in controlling the cell cycle, maintaining genomic integrity, controlling subcellular transport, and regulating transcriptional mechanisms. SIZ1 (SUMO ligase) is a SUMO E3 ligase that mediates the coupling with ICE1, enhances the stability of ICE1 at low temperatures, and helps to induce the expression of *CBF3*/*DREB1A*, positively regulating the cold tolerance of plants [79]. Based on the above review of relevant research on plant responses to low-temperature stress signals, the molecular mechanism pattern of plant signal transduction pathways in response to low-temperature stress is summarized (Figure 1).

### 3.5. Non-Coding RNA Regulation

In the complex physiological processes of plants responding to low-temperature stress, non-coding RNAs (ncRNAs) play a crucial regulatory role. Non-coding RNAs are a class of RNA molecules that do not encode proteins but have important functions in multiple aspects such as gene expression regulation. They mainly include long non-coding RNAs (lncRNAs), microRNAs (miRNAs), and short interfering RNAs (siRNAs) [97].

#### 3.5.1. Mechanisms of miRNAs in Regulating Plant Tolerance to Low Temperature

miRNAs usually regulate gene expression at the post-transcriptional level by binding to target mRNAs through complementary base pairing. Under low-temperature stress, the expression levels of specific miRNAs in plants change, thereby regulating the expression of their target genes. For example, the expression level of miR169 decreases after low temperature treatment. Its target genes are members of the nuclear factor Y (NF-YA) family. As a transcription factor, NF-YA is involved in regulating the expression of many genes related to the low temperature response. The decrease in miR169 expression leads to an increase in NF-YA expression, which activates a series of low temperature response genes and enhances plant tolerance to low temperature [98]. Some other miRNAs enhance plant resistance by inhibiting genes that are sensitive to low temperature. For instance, the expression of miR319 is upregulated under low-temperature stress. It targets and regulates some genes involved in cell cycle and growth regulation. By inhibiting the expression of these genes, the plant growth rate slows down, and more energy and resources are allocated to coping with low-temperature stress [99]. miRNAs can participate in plant hormone signal transduction pathways and indirectly regulate the plant’s response to low temperature. For example, miR160 regulates auxin signal transduction by targeting auxin response factors (ARFs). Under low-temperature stress, changes in the miR160-ARF module affect plant growth and development and also regulate plants’ adaptability to low temperature. In addition, the miR156-SPL module is also involved in regulating the transition of plant growth stages and stress responses. Under low-temperature stress, the regulation of this module helps plants adjust their growth strategies and improves their low temperature tolerance [100].

#### 3.5.2. Mechanisms of lncRNA in Regulating Plant Tolerance to Low Temperature

lncRNAs can act as competing endogenous RNAs (ceRNAs) and regulate gene expression by sequestering miRNAs. Under low-temperature stress, some lncRNAs bind to specific miRNAs, relieving the inhibitory effect of miRNAs on their target mRNAs. For example, a certain lncRNA can bind to miR172, and miR172 inhibits the expression of AP2-like transcription factor genes related to flowering and the low temperature response. When the lncRNA sequesters miR172, the AP2-like transcription factor genes are expressed, thereby regulating downstream genes related to low temperature tolerance and improving the plant’s adaptability to low temperature [101].

lncRNAs can interact with chromatin-remodeling complexes, transcription factors, etc., affecting the structure of chromatin and the transcriptional activity of genes. In the response to low-temperature stress, some lncRNAs can recruit histone-modifying enzymes to the promoter regions of specific genes, changing the modification state of histones (such as methylation, acetylation, etc.) and thereby regulating gene transcription. For example, certain lncRNAs interact with histone methyltransferases, causing specific methylation modifications of histones in the promoter regions of low temperature response genes, which promotes the transcription of these genes and enhances the plant’s low temperature tolerance [102].

#### 3.5.3. Mechanisms of siRNA in Regulating Plant Tolerance to Low Temperature

siRNAs mainly regulate gene expression through the RNA interference (RNAi) mechanism, specifically degrading target mRNAs. Under low-temperature stress, some siRNAs related to the low temperature response produced in plants can recognize and bind to specific mRNAs, guiding nucleases to cleave and degrade them. For example, certain siRNAs target and regulate genes related to plant hormone signal transduction, antioxidant defense, and other pathways. By regulating the expression levels of these genes, the hormonal balance and the stability of the antioxidant defense system in plants are maintained, helping plants better cope with low-temperature stress [103]. siRNAs can also participate in the regulation of DNA methylation. In the response to low-temperature stress, some siRNAs can guide DNA methyltransferases to add methyl groups to the promoter regions of specific genes, resulting in gene silencing or changes in expression levels. This DNA methylation modification can regulate the expression of genes related to low temperature tolerance, thereby affecting the plant’s adaptability to low temperature. For example, through the DNA methylation mediated by siRNAs, the expression of some genes involved in regulating cell membrane fluidity and the synthesis of osmoregulatory substances is regulated, improving plants’ ability to survive in a low-temperature environment [104].

### 3.6. Epigenetic Mechanism Responses to Stress

Low-temperature stress can trigger a variety of epigenetic modification changes, mainly involving DNA methylation, histone modification, chromatin remodeling, and non-coding RNA regulation [105].

#### 3.6.1. DNA Methylation

DNA methylation is the process by which methyl groups are added to specific DNA regions under the action of DNA methyltransferases. Under low-temperature stress, an increase in the DNA methylation level in the promoter regions of some genes can impede the binding of transcription factors to DNA, thereby suppressing the expression of related genes. For example, certain genes involved in plant growth and development that may be unfavorable for plant cold resistance under low temperatures are suppressed through methylation modification. This allows plants to allocate more resources to physiological processes. These processes are related to cold resistance. Conversely, the methylation levels of some genes related to the low temperature response decrease, which promotes gene transcription. This enables plants to synthesize more proteins required to cope with low-temperature stress. An example of this are cold-responsive genes (COR genes), whose expression products help improve the freezing resistance of plant cells and regulate the intracellular osmotic pressure [106,107], etc.

#### 3.6.2. Histone Modifications

Histones can undergo various modifications, such as methylation, acetylation, phosphorylation, etc. These modifications can alter the structure and function of chromatin, thereby influencing gene expression. Generally, histone acetylation makes the chromatin structure more relaxed, increases the accessibility of genes, and promotes gene transcription [108].

Under low-temperature stress, the histone acetylation level in the regions of some cold resistance related genes increases. This enables these genes to be transcribed and expressed smoothly, thus enhancing the plant’s cold resistance. For example, the acetylation modifications of histones H3 and H4 are associated with the activation of some low-temperature-induced genes [109].

In addition, the modification sites and degrees of histone methylation have different effects on gene expression. Methylation at certain sites may promote gene expression, while methylation at other sites may inhibit gene expression. During the low-temperature stress response, the histone methylation pattern in specific gene regions changes to regulate the expression of related genes and participate in the plant’s adaptation to the low temperature environment [110,111].

#### 3.6.3. Chromatin Remodeling

Chromatin-remodeling complexes can regulate the structure of chromatin and the accessibility of genes by changing the position, the composition of nucleosomes on DNA, or their interaction with DNA. Under low-temperature stress, chromatin-remodeling complexes are recruited to specific gene regions, making the originally tightly packed chromatin structure more relaxed. This allows the transcription machinery to more easily access the gene promoter regions, promoting the transcription of low-temperature response genes [112].

#### 3.6.4. Non-Coding RNA Regulation

Non-coding RNAs include microRNAs (miRNAs), long non-coding RNAs (lncRNAs), etc., and they play important roles in the low-temperature stress response. miRNAs can cause the degradation of target mRNAs or inhibit their translation process through complementary pairing with target mRNAs. Under low-temperature stress, the expression levels of some miRNAs in plants change [113]. LncRNAs can play regulatory roles at multiple levels, such as the transcriptional and post-transcriptional levels. Some lncRNAs can interact with DNA, RNA, or proteins to regulate the state of chromatin and gene expression. During the low-temperature stress response, specific lncRNAs may form RNA-DNA or RNA–protein complexes with related genes, affecting the transcription and translation of genes and thus participating in the plant’s adaptation process to low temperatures [114].

These epigenetic regulatory pathways do not function independently. They are interrelated and interact with each other, forming a complex regulatory network. Together, they regulate the gene expression and physiological responses of plants under low-temperature stress, helping plants adapt to low-temperature environments. Moreover, one of the most remarkable features of epigenetic systems is their ability to transfer adaptive traits across generations. This process, known as intergenerational adaptation, involves the inheritance of stress-induced epigenetic changes, enabling offspring to better withstand similar stressors. Extending beyond the immediate generation, transgenerational adaptation ensures the persistence of these traits over multiple generations, promoting long-term survival in changing environments. These mechanisms highlight the pivotal role of epigenetics in shaping the evolutionary trajectories of plants [115].

## 4. Research Progress on Improving Plant Cold Resistance by Genetic Engineering

Affected by the greenhouse effect, extreme weather occurs frequently. In agricultural production, crops often suffer from low temperatures, which disrupt physiological and developmental processes, lead to poor crop growth, and eventually cause low fruit setting rates, reduced yields, and even plant death [115]. With the continuous increase in the global population, the demand for various crop products, especially food, is growing. Using genetic engineering techniques to improve plant stress resistance and cultivate varieties with superior cold resistance to harvest more crop yields significant for agricultural development and socioeconomic growth [116].

Studies have shown that overexpression of *bZIP73* and *bZIP71* in rice, which form heterodimers, can inhibit ABA biosynthesis and promote the transport of soluble sugars from anthers to pollen, thereby enhancing the cold resistance of rice during the reproductive stage and ultimately increase fruit setting rates and grain yield [117]. Transgenic rice overexpressing *DREB1A* can increase the content of osmoprotectants such as proline and various sugars, enhancing its tolerance to drought, high salinity, and low-temperature stress [100]. Expression of the R2R3-MYB transcription factor family member *OsMYB2* in rice enhances plant tolerance to salt, low temperature, and dehydration stress, improves seed germination rates, accumulates more soluble sugars and free proline, promotes osmoregulation, and enhances rice cold resistance [118]. Overexpression of the NAC transcription factor family member *SNAC2* gene helps rice enhance cell membrane stability, improve tolerance to osmotic stress, and produce a series of products such as peroxidases, ornithine aminotransferases, lysine ketoglutarate reductases, heavy metal-related proteins, sodium/hydrogen exchangers, heat shock proteins, and GDSL-like lipases, ultimately resulting in significantly improved tolerance to cold, salt, and dehydration stress [119]. Studies have shown that the small nuclear GTPase RAN2 primarily mediates the exchange of GTP in the nucleus and GDP in the cytoplasm in plants. Therefore, overexpression of the RAN2 gene in rice can promote the normal export of microtubulin in plant cell nuclei, thereby maintaining stable cell division and improving rice cold resistance [120].

Research shows that in maize, overexpressing *ZmbZIP4* significantly increases the number of lateral roots in transgenic plants compared with wild-type plants. The main roots of plants become longer, their root systems are improved, and many stress response genes and abscisic acid synthesis-related genes are generated to enhance the plant’s ability to resist abiotic stress [24]. Overexpressing the *ZmMYB31* gene increases the expression of relevant cold stress genes in maize and reduces ion leakage, ROS content, and low-temperature photoinhibition phenomena caused by cold stress, thus playing a positive regulatory role in cold and peroxide stress [121]. The *ZmICE1* gene can inhibit the expression of asparagine synthetase, a Glu/Asn biosynthesis gene, to reduce ROS production, and it can directly regulate the expression of *DREB1*. Moreover, the combination of the *ZmICE1* promoter and the positive regulator *ZmMYB39* can significantly increase the cold resistance of maize. Over expressing *ZmICE1* has no obvious negative impact on maize yield-related traits, indicating that overexpressing the *ZmICE1* gene can not only cultivate new cold-tolerant maize varieties but also will not affect maize yield [122].

Research progress on transgenic wheat and barley shows that cloning the *HDZI* 3 and *HDZI* 4 promoters from the HD-Zip I gene of wheat successfully optimizes the expression of the *TaCBF5L* and *TaDREB3* genes in transgenic wheat and barley under abiotic stress, which can improve the cold resistance of transgenic plants and reduce the negative impact of transgenics on plant development and grain yield [123]. Cloning the *WRKY71* gene promoter of rice and the Cor39 gene promoter of wheat can optimize the expression of *TaDREB3* and construct transgenic barley lines. Compared with wild-type plants, overexpressing *TaDREB3* significantly improves the frost resistance of barley and increases the expression of cold-responsive genes while having no negative impact on plant characteristics and grain yield. Isolating the DREB/CBF gene *TaRAP2.1L* from wheat as a stress-responsive transcriptional repressor, the generated *TaRAP2.1L* mutant plants can activate the expression of downstream DREB/CBF genes, thereby enhancing the frost and drought resistance of wheat while having no negative impact on the growth and production of wheat [124].

Overexpressing the cold-regulated gene *LeCOR413PM2* (*Cold Regulated 413 Plasma Membrane 2*) in tomatoes can reduce damage to plant cell membranes, decrease the accumulation of ROS in plants and the photoinhibition of PS II, and maintain the high activity of antioxidant enzymes and the content of osmotic-regulating substances in the body, thereby improving the cold tolerance of transgenic tomato plants [125]. The HY5-MYB15-CBFs transcriptional cascade has been proven by research to play an important role in the cold response of tomatoes. When the *HY5* or *MYB15* gene is overexpressed in tomato, the expression of *CBF1*, *CBF2*, and *CBF3* can be activated, thereby enhancing the cold tolerance of tomato crops [126]. Overexpressing the brassinosteroid receptor *SlBRI1* (*Brassinosteroid Insensitive 1*) in tomatoes can reduce the accumulation of MDA and ROS and increase the activities of SOD, POD, and CAT. Moreover, in transgenic plants overexpressing SlBRI1, the expression levels of *ICE1*- and *CBF*-related genes are significantly increased, indicating that SlBRI1 increases the tolerance of tomato to cold stress by affecting the transcriptional level of the ICE1-CBF-COR pathway and positively influencing the ROS scavenging system, the photoinhibition of the photosystem, and the biosynthesis and signal transduction of phytohormones [127]. *SlDREB3* expression in tomatoes can help tomatoes reduce the ROS accumulation and cell damage caused by freezing under cold stress. At the same time, it can also improve the tolerance of transgenic lines to low-temperature stress by activating the expression of SlLEA genes [128].

## 5. Future Directions

### 5.1. In-Depth Analysis of Molecular Regulatory Mechanisms

#### 5.1.1. Refinement of Epigenetic Regulatory Networks

Although multiple epigenetic modifications, such as DNA methylation, histone modifications, and non-coding RNA regulation, are known to be involved in plant responses to low temperature, the details of the synergistic effects and regulatory networks among these modifications remain unclear. For example, the questions of how different types of histone modifications interact with DNA methylation to jointly regulate the expression of low-temperature-related genes and how non-coding RNAs accurately recognize target sites and cooperate with other epigenetic factors to regulate gene expression require investigation [129].

#### 5.1.2. Precise Regulation of Transcription Factors and Downstream Genes

Many transcription factors play crucial roles in plant responses to low temperature. However, the questions of how they accurately recognize and bind to the promoter regions of downstream target genes and how they recruit the transcriptional machinery to initiate the transcription process still need in-depth study. In addition, the interaction networks among transcription factors and how they integrate multiple signals to regulate plant responses to low temperature also await further clarification [129].

#### 5.1.3. Role of Post-Translational Modifications of Proteins

Post-translational modifications of proteins, such as phosphorylation, ubiquitination, and SUMOylation, may play important roles in the signal transduction process of plant responses to low temperature. Currently, our understanding of how these modifications regulate the activity, stability, and localization of proteins and of how they affect plant tolerance to low temperature is limited [130].

### 5.2. Completion of the Signal Transduction Pathway for Low Temperature Responses

#### 5.2.1. Mechanisms of Signal Molecules

In addition to common plant hormones (such as abscisic acid and ethylene) that play roles in plant responses to low temperature, the mechanisms of action of some new signal molecules (such as reactive oxygen species and nitric oxide) need further study. For example, the questions of how these signal molecules are generated, transmitted, and amplified within cells and how they intersect and integrate with other signal pathways require clarification [131].

#### 5.2.2. Mechanisms of Low Temperature Sensing by the Cell Membrane

The cell membrane is an important site for plants to sense low temperature. However, the specific molecular mechanisms by which the cell membrane senses low temperature signals and converts this physical signal into an intracellular chemical signal (such as changes in calcium ion concentration) are still unclear. In depth research on this process will help to reveal the initiation mechanism of plant responses to low temperature [131].

### 5.3. Integrated Analysis of Multi-Omics

#### 5.3.1. Integration of Transcriptomic, Proteomic, and Metabolomic Data

Through the combined analysis of multi-omics technologies, we can comprehensively understand the dynamic changes in gene expression, protein abundance, and metabolite levels in plants under low-temperature stress, thereby constructing a more complete regulatory network for low temperature responses. For example, by integrating transcriptomic and proteomic data, we can better understand the regulatory relationship between gene transcription and protein translation; incorporating metabolomic data into the analysis can reveal the laws of material and energy metabolism during low-temperature responses [132].

#### 5.3.2. Spatiotemporal Multi-Omics Research

Conducting spatiotemporal multi-omics research, that is, performing multi-omics analysis at different time points and in different plant tissue parts, helps to gain in depth insights into the dynamic process and tissue specific regulatory mechanisms of plant responses to low temperature. This can help us clarify the functional division and cooperation of different tissues under low-temperature stress [132].

### 5.4. Interaction Between Plant Low Temperature Responses and Other Environmental Factors

#### 5.4.1. Synergistic Effects of Low Temperature with Drought, Salt Stress, etc.

In the natural environment, plants often face multiple stress conditions simultaneously, such as the co-occurrence of low temperature with drought or salt stress. Studying the synergistic mechanisms among these different stresses helps to reveal the adaptation strategies of plants in complex environments. For example, understanding how plants integrate low temperature and drought signals, and how the corresponding regulatory mechanisms interact with each other, is of great significance for breeding crop varieties that can adapt to multiple stresses [133].

#### 5.4.2. Interaction Between Low Temperature and Microorganisms

The plant rhizosphere microbial community can affect plant growth and stress resistance. Studying the interaction between plants and rhizosphere microorganisms in low-temperature environments, such as the processes through which microorganisms help plants improve low temperature tolerance and through which plants recruit beneficial microorganisms by regulating the rhizosphere microenvironment, will provide new ideas for using microorganisms to enhance plant low temperature adaptability [133].

## 6. Conclusions

Low-temperature stress, including chilling and freezing injuries, negatively impacts plant growth, particularly in tropical and temperate regions. Cold-acclimated plants develop mechanisms to enhance freezing tolerance by regulating photosynthesis, metabolism, and protein pathways, producing osmotic regulators and antioxidants, and modulating hormones. The cell membrane plays a critical role in maintaining cellular function under stress, with cold-resistant plants exhibiting higher membrane lipid unsaturation, preserving fluidity and normal metabolism. Additionally, plants accumulate osmotic regulators like proline and soluble sugars to maintain osmotic balance and protect against dehydration. Reactive oxygen species (ROS) accumulation can cause cellular damage, but antioxidants mitigate this, while plant hormones like ABA, auxin, and gibberellins aid in cold adaptation. In addition, key genes and regulatory pathways, such as the ICE1-CBF-COR cascade, help plants adapt to low temperatures, ensuring survival under cold stress through complex molecular responses.
